# Antimicrobial Sensitivity Testing of *Mycoplasma bovis* Isolates Derived from Western Canadian Feedlot Cattle

**DOI:** 10.3390/microorganisms8010124

**Published:** 2020-01-16

**Authors:** Murray Jelinski, Andrea Kinnear, Karen Gesy, Sara Andrés-Lasheras, Rahat Zaheer, Scott Weese, Tim A. McAllister

**Affiliations:** 1 Western College of Veterinary Medicine, University of Saskatchewan, Saskatoon, SK S7N 5B4, Canada; andrea.kinnear@usask.ca (A.K.); karen.gesy@usask.ca (K.G.); 2 Lethbridge Research and Development Centre, Agriculture and Agri-Food Canada, Lethbridge, AB T1J 4B1, Canada; sara.andreslasheras@canada.ca (S.A.-L.); rahat.zaheer@canada.ca (R.Z.); tim.mcallister@canada.ca (T.A.M.); 3 Ontario Veterinary College, University of Guelph, Guelph, ON N1G 2W1, Canada; jsweese@uoguelph.ca

**Keywords:** beef, feedlot, *Mycoplasma bovis*, antimicrobial, resistance, susceptibility, Canada

## Abstract

*Mycoplasma bovis* is particularly adept at evading the immune system, resulting in chronic infections of the lungs and joints of feedlot cattle. The chronicity of the lesions results in prolonged antimicrobial therapy, possibly exacerbating antimicrobial resistance. This cross-sectional study generated in vitro antimicrobial susceptibility testing (AST) data on 211 *M. bovis* isolates recovered from 159 healthy, diseased, and dead cattle, spanning the period of 2006–2018. Nine antimicrobials commonly administered to western Canadian feedlot cattle were assessed. The data were analyzed with non-parametric statistical tests with a level of significance of *p* < 0.05 (two-tailed). Minimum inhibitory concentration (MIC) values tended to increase between the isolates from healthy versus dead cattle and over time (2006–2018). Isolates from dead versus healthy cattle were more likely to be resistant to tulathromycin, gamithromycin, tylosin and enrofloxacin. There was no difference in the distributions of the MICs generated from the isolates recovered from the lungs and joints (*p* ≥ 0.124) and the lungs and deep nasal passages (*p* ≥ 0.157) of the same animals.

## 1. Introduction

Canada’s beef industry is comprised of two main sectors, cow–calf and feedlot. Calves are born in the spring of the year and then raised on pasture until weaned in the fall. Most are transported to an auction where they are commingled, sorted, and sold into feedlots. Commingling and transport predisposes cattle to developing bovine respiratory disease (BRD) or “Shipping Fever,” which is the leading cause of morbidity and mortality in weaned beef calves. The most common bacterial pathogens associated with BRD are *Mannheimia haemolytica*, *Pasteurella multocida*, *Histophilus somni* and *Mycoplasma bovis* [1]. Furthermore, the polymicrobial nature of BRD makes it difficult to ascribe a specific pathogen(s) to an individual case of BRD, since affected animals typically present non-specific clinical signs such as anorexia, depression, coughing and fever. The epidemiology of BRD is well known, with cases occurring within 6–10 day of entry into the feedlot [2]. Therefore, antimicrobial metaphylaxis is routine for on-arrival cattle that are deemed a high risk of developing BRD [3,4]. A study of antimicrobial usage (AMU) in western Canadian feedlots for the years 2008–2012 found that tetracyclines and macrolides were the classes of drugs that were most commonly administered parenterally. Furthermore, 39% of all cattle arriving to the feedlots were categorized as high risk; 95% of these cattle received parenterally administered BRD metaphylaxis, while 59% of the low risk cattle also received parenterally administered metaphylaxis [5]. Macrolides are the primary antimicrobials that are administered to high risk cattle, whereas tetracyclines are administered to low risk cattle. Fluoroquinolones and ceftiofur are used sparingly, comprising <1% of the total amount of AMU of medically important antimicrobials used in feedlots.

There is some debate as to whether *M. bovis* is a primary pathogen, a secondary invader, or a predisposing factor for other BRD bacterial agents [6,7,8,9]. However, it is accepted that *M. bovis* can be routinely recovered from the lungs of cattle with subacute-to-chronic BRD [1,6,10,11]. Furthermore, the finding of chronic caseonecrotic bronchopneumonia on postmortem examination is considered pathognomonic of an *M. bovis* infection [6,12,13]. Cattle with a concurrent septic polyarthritis and caseonecrotic pneumonia are given a diagnosis of chronic pneumonia and polyarthritis syndrome (CPPS) [14], which was previously known as the ‘pneumo-arthritis syndrome of calves’ [15]. The chronicity of mycoplasmosis is salient because diseased cattle receive repeated antimicrobial therapies [10] that may lead to increased antimicrobial resistance (AMR) within bacterial communities.

There are four Canadian cattle studies of interest wherein antimicrobial susceptibility testing (AST) data were generated from *M. bovis* isolates. Two were conducted on *M. bovis* isolates that had been derived from cattle in eastern Canada. However, in both studies, the isolates were primarily from dairy cattle and the most contemporaneous isolates were from 2009 [16,17]. The third study provides AST results from 51 *M. bovis* isolates recovered from western Canadian feedlot cattle; however, all isolates originated from one feedlot [18]. In addition, the isolates predated some of the newer macrolide products currently used to treat BRD. More recently, AST data were generated from 226 *M. bovis* isolates that were derived from 211 western Canadian feedlot cattle [19]. However, the majority of tested antimicrobials are not routinely used in feedlot cattle (aminoglycosides, lincosamides, and pleuromutilin) or have no antibacterial activity to mycoplasmas (β-lactams, trimethoprim, and sulphonamides) [20,21].

The objective of this retrospective cross-sectional study was to conduct AST on 211 *M. bovis* isolates that were recovered over a multi-year period (2006–2018) from western Canadian feedlot cattle. Isolates were collected from multiple feedlots, three anatomical sites (i.e., nasopharynx, lung, and joint) and from healthy, diseased, and dead cattle. A customized 96-well broth microdilution test plate was used for AST of nine antimicrobials most commonly administered to cattle in western Canadian feedlots for the metaphylaxis and treatment of BRD.

## 2. Materials and Methods

### 2.1. Sample Collection and Handling

This study was approved by the University of Saskatchewan’s Animal Research Ethics Board (Protocols 20070023 and 20170021) and the Lethbridge Research Centre’s Animal Care Committee (Protocol 1641).

This cross-sectional study obtained clinical samples from cattle of varying health statuses (healthy, diseased, and dead), from different anatomical regions (deep nasopharyngeal passages, lungs and joints), and over a multi-year period (2006–2018). The long timeframe coupled with the involvement of multiple private veterinary practices resulted in minor changes to the sampling procedures, culturing methods, and *M. bovis* isolation techniques. However, all antimicrobial susceptibility testing (AST) was standardized, with samples being batch processed by two individuals over a six month period.

Diseased cattle were identified by the feedlots’ trained personnel. A diagnosis of BRD was based on a constellation of clinical signs such as depression, nasal discharge, elevated respiratory rate, anorexia and fever. Guarded uterine swabs (Reproduction Resources, Walworth, WI, USA) were used to obtain deep nasopharyngeal (DNP) samples from healthy animals and from cattle who showed clinical signs of BRD (diseased). Immediately following sampling, the swabs were placed in Ames media (Mai, Ames Media, Product Number 49203, Spring Valley, WI, USA).

Three feedlot veterinary practices, who manage the health programs of the majority of feedlot cattle in Canada, were recruited to provide clinical material from necropsied animals. A purposive sampling strategy was followed with the veterinary practices being provided sampling kits and written standard operating procedures for selecting and sampling cases. The target population was animals that died or were euthanized as a result of *M. bovis* pneumonia or chronic pneumonia and polyarthritis syndrome (CPPS). The lungs had to have a gross pathology that was consistent with chronic bronchopneumonia and/or a caseonecrotic pneumonia. If the animals had concurrent septic arthritis, then, whenever possible, the joint(s) was sampled by swabbing, aspirating joint fluid into a syringe, or excising synovial tissue. Veterinarians were instructed to excise a minimum 3 × 3 cm block of healthy and diseased lung tissue. Because of the varying distance to the laboratory and the random nature of the occurrence of the cases, samples were frequently stored at −20 °C until they were batch shipped.

The animal’s feedlot record accompanied the sample and provided the date of death, number of days on feed (DOF), and treatment history. The following metadata were also recorded: date of sampling, type of sample (swab, tissue, or joint fluid), anatomical region (nasopharynx, lung, or joint), and health status (healthy, diseased, or dead). Because most feedlot cattle enter the feedlot in the fall of the year and are carried into the following year, sampling was defined by production year (cohort) [5]. For example, the 2006 production year (cohort) included samples obtained between 1 November 2006 and 30 June 2007.

### 2.2. Mycoplasma spp. Isolation

The DNP swabs and swabs taken from fresh cut tissue surfaces (lung and joint) were used to inoculate either Hayflick’s (prepared in-house) or PPLO (pleuropneumonia-like organisms) broth (BD Difco, Fisher Scientific, Waltham, MA, USA). Samples collected from 2006 to 2008 and were cultured with Hayflick’s media, while all subsequent samples were cultured with neat PPLO media supplemented with 20% (*v*/*v*) horse serum (Invitrogen, Fisher Scientific) and 10 g/L of yeast extract (BD Diagnostic Systems, Fisher Scientific). Further media supplementation is indicated where applicable. The cultures were filtered at 0.45 and 0.20 µm (Basix, VWR International, Radnor, PA, USA) prior to inoculating in a PPLO broth containing 0.05% thallium (I) acetate, 500 U/mL penicillin G, and 0.5% sodium pyruvate (Sigma-Aldrich, St. Louis, MO, USA. Sodium pyruvate was added during initial growth to further enrich for *M. bovis*. All broth and agar plate incubations were conducted at 37 °C with 5% CO_2_ and 75% humidity. Cultures with visible growth were streaked onto PPLO agar (BD Difco, Fisher Scientific) with 0.05% thallium (I) acetate and 500 U/mL penicillin G, and they were then incubated for 3–6 day. An isolated putative *M. bovis* colony, based on the characteristic “fried-egg” morphology, was picked, plated, and incubated for 72 h. Up to three individual colonies were selected and inoculated separately into a PPLO broth with 0.05% thallium (I) acetate and 500 U/mL penicillin G. Cultures were incubated for 48 h and stored in a PPLO broth with glycerol (20%, *v*/*v*) at −80 °C. One individual culture was processed for DNA extraction and identification. After confirmation of the isolate as *M. bovis*, the culture was subjected to AST.

### 2.3. Species Identification

Cultures were centrifuged (10 min at 2500× *g*), and the DNA was extracted with a GenElute Bacterial Genomic DNA Kit (Sigma-Aldrich). The manufacturer’s instructions for Gram-negative bacterial DNA extraction were followed with the final elution buffer replaced with 10 mM Tris (pH 8.5). To confirm a pure culture of *Mycoplasma bovis*, DNA excision repair gene *uvrC* and 16S ribosomal (rRNA) genes were amplified by using specific primers, as per previous reports [22,23]. The PCR product corresponding to the 16S rRNA gene was purified with the QIAquick PCR Purification Kit (Qiagen, Venlo, Netherlands) and sent to Macrogen (Seoul, South Korea) for Sanger sequencing. The consensus sequence was generated with Staden (version 1.6-r) and verified as *M. bovis* via the NCBI Blastn Suite.

### 2.4. Antimicrobial Susceptibility Testing

Antimicrobial susceptibility testing was performed by using a customized 96-well Sensititre plate (Trek Diagnostics, Oakwood, GA, USA) with the following antimicrobials: enrofloxacin (ENRO), gamithromycin (GAM), tulathromycin (TUL), tildipirosin (TIP), tilmicosin (TIL), tylosin tartrate (TYL), florfenicol (FFN), oxytetracycline (OXY), chlortetracycline (CTET), and penicillin (PEN). Serial two-fold dilutions were prepared as follows: ENRO, 0.12–128 µg/mL; TIP, 0.12–128 µg/mL; GAM, 0.25–256 µg/mL; TUL, 0.25–256 µg/mL; TIL, 1–256 µg/mL; TYL, 1–128 µg/mL; FFN, 0.25–256 µg/mL; OXY, 0.5–256 µg/mL; and CTET, 1–256 µg/mL. PEN (2–8 µg/mL) served as a control. Growth was assessed by using a color redox indicator, alamarBlue (Invitrogen, Fisher Scientific), based on a blue-to-pink color change.

For AST, the reserve culture was inoculated into the PPLO broth with 0.5% sodium pyruvate and incubated for 72 h. Following incubation, the actively growing broth cultures of isolates were subcultured into a neat PPLO broth and incubated for 24 h. Next, the optical density (OD) at 450 nm was determined by using a NanoDrop One Spectrophotometer (Fisher Scientific) and cultures were normalized to an OD_450_ = 0.1. Cultures were further diluted up to 10× in neat PPLO media prior to preparation of the inoculum. This inoculum (culture: media, 1:50) was prepared in a 20% alamarBlue solution in neat PPLO media and added to each well according to the manufacturer’s procedure (Trek Diagnostics, Thermo Fischer Scientific, Oakwood, OH, USA). This provided a final concentration of 10^3^ to 5 × 10^5^ CFU/mL in a 10% alamarBlue solution (as per manufacturer’s instructions). The plates were sealed with a CO_2_ permeable film and incubated for 48–72 h. Minimum inhibitory concentrations (MIC) were visually determined at 48 and 72 h and reported as per the Clinical and Laboratory Standards Institute (CLSI) guidelines [24].

If growth was observed in the positive control wells (no antibiotics), then the MIC values for that isolate were accepted. The MIC was defined as the lowest concentration of antimicrobial that prevented visible growth of the inoculated *M. bovis* culture. A *M. bovis* reference strain (*Mycoplasma* bovis ATCC^®^ 25523™) was tested five times for quality control.

### 2.5. Breakpoint Interpretation Guidelines

The CLSI has no approved MIC breakpoint values for the mycoplasmas of livestock, but it has established interpretive breakpoints for human mycoplasmas and three respiratory pathogens of cattle: *Mannheimia haemolytica*, *Pasteurella multocida*, and *Histophilus somni* [25,26]. The suggested breakpoint interpretations provided herein were derived from three sources: CLSI guidelines [26], previous publications, and the MIC data generated in the current study. The Clinical and Laboratory Standards Institute (CLSI) has assigned the same breakpoints to *Mannheimia haemolytica*, *Pasteurella multocida*, and *Histophilus somni* for TUL, GAM, ENRO, FFN, and OXY [26]. Only *Mannheimia haemolytica* has breakpoints for TIL. All breakpoints were adopted for this study. Furthermore, the OXY breakpoints were applied to CTET. The suggested intermediate and resistance interpretative breakpoints for TIP are lower for *Mannheimia haemolytica* (8 and ≥16 µg/mL) than for *Pasteurella multocida* and *Histophilus somni* (16 and ≥32 µg/mL). The lower set of breakpoints was chosen because they did not bisect the unimodal MIC distributions. No CLSI breakpoints exist for TYL. However, *M. bovis* isolates with an MIC of ≤4 µg/mL have no point mutations associated with resistance, whereas single point mutations in one or more alleles are associated with MIC values of 8–32 µg/mL [27]. Therefore, ≥8 µg/mL was used as the breakpoint for TYL. 

Epidemiological cut-off values (ECV) were assigned to antimicrobials with a bi-modal MIC distribution.

### 2.6. Data Analysis

Data were captured in a commercial spreadsheet (Microsoft Excel version 12; Microsoft Corporation, Redmond, Washington, WA, USA) and then imported into a statistical program for analyses (IBM SPSS Statistics version 26, IBM Corporation, Armonk, NY, USA). The MIC data of each antimicrobial were tabulated, graphed, and visually assessed. The MIC data were considered ordinal data and analyzed by using non-parametric test statistics. The Kruskal–Wallis test statistic with a Bonferroni corrected post-hoc analyses and the Mann–Whitney test statistic were used to assess for differences in MIC distributions. The Wilcoxon signed-rank test was used to analyze the MIC distributions of the paired lung–joint and paired DNP–lung isolates. Mean ranks provided a measure of effect. Spearman’s correlation assessed whether the antimicrobials’ MICs were correlated, where Spearman’s correlation coefficient rho (ρ) value of > 0.8 was considered a strong correlation, 0.6–0.8 as moderate, 0.3–0.5 as fair, and 0.1–0.2 as poor. Suggested susceptibility (S), intermediate (I), and resistance (R) breakpoints were provided, and the Chi-square statistic was used to analyze for differences in the percentage of resistant isolates by health status and over time. The level of significance for all statistical tests was *p*-value < 0.05 (two-tailed).

## 3. Results

### 3.1. Sample History

*Mycoplasma bovis* isolates were derived from a broad cross-section of cattle in time and place. Of the 211 clinical isolates, 14 were recovered from cattle imported from Idaho, USA, and the remaining 197 came from cattle raised in western Canada and sold to feedlots via auctions or directly from the ranch. Feedlot records were used to associate the cattle to a feedlot and cohort, where a cohort was a group of animals in the same feedlot at the same time. The 211 isolates were recovered from cattle that represented 31 feedlots, 40 cohorts, and a 12 year time interval (Appendix A).

Table 1 is a cross tabulation of the *M. bovis* isolates by anatomical region, health status, and production year (placement cohort). The 211 clinical isolates were recovered from 159 different animals, 111 of which yielded one isolate (66 DNP, 36 lung, nine joint), 36 yielded a pair of lung–joint isolates (*n* = 72), eight yielded a pair of lung–DNP isolates (*n* = 16), and four animals provided a set of DNP, lung, and joint isolates (*n* = 12). Thus, 40 animals provided paired lung–joint isolates (*n* = 80 isolates), and 12 animals provided paired DNP–lung isolates (*n* = 24 isolates). These pairings were separately analyzed to determine if the MIC distributions differed by anatomical location.

Forty-three healthy cattle from 14 cohorts yielded DNP isolates. The 14 American cattle were all healthy at the time of DNP sampling, and all were sampled in 2017 and 2018. The mean (median) DOF at sampling was 14 DOF (0 DOF) with a range of 0–140. Over half (58.1%; *n* = 25) were sampled on-arrival (0 DOF). 

Nineteen DNP isolates came from diseased cattle, representing five cohorts, with a mean (median) DOF at sampling of 55 DOF (21 DOF) and range of 0–150. Ten (52.6%) of the diseased cattle were ≤21 DOF at the time of sampling.

Most isolates (*n* = 149) came from 122 cattle that died of pneumonia between 9 and 217 DOF, with mean (median) of 47 DOF (42 DOF). These mortalities came from 24 cohorts of cattle placed in 20 different feedlots, with 76 (51.0%) isolates coming from four cohorts within one feedlot over a four-year period.

### 3.2. Antimicrobial Susceptibility Testing

The minimum inhibitory concentration (MIC) values for the *M. bovis* reference strain (ATCC^®^ 25523™) were consistent across the AST processing period: TUL 0.25 µg/mL, GAM 8–16 µg/mL, TIL 1 µg/mL, TIP 4–8 µg/mL, TYL 1–2 µg/mL, ENRO 0.12–0.25 µg/mL, FFN 1–2 µg/mL, OXY 1–2 µg/mL, and CTET 1 µg/mL.

**Figure 1:**
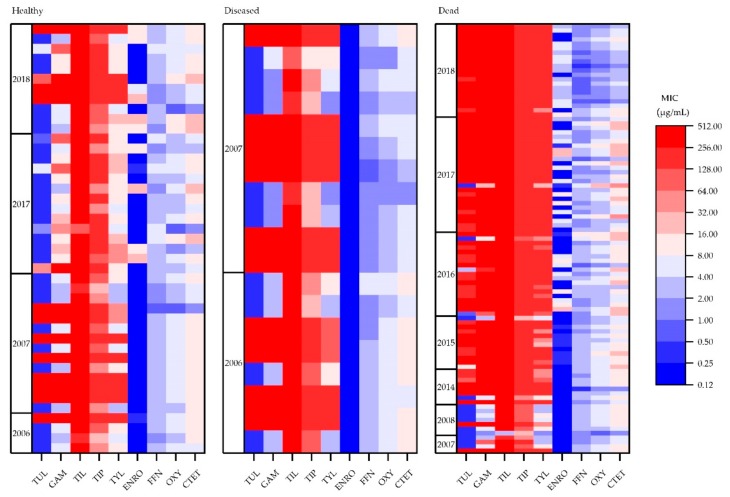
Minimum inhibitory concentration distribution for *M. bovis* isolates from healthy (*n* = 43), diseased (n = 19), and dead (*n* = 97) cattle (*n* = 159). Tulathromycin (TUL; 0.25–256 µg/mL), gamithromycin (GAM; 0.25–256 µg/mL), tilmicosin (TIL; 1–256 µg/mL), tildipirosin (TIP; 0.12–128 µg/mL), tylosin tartrate (TYL; 1–128 µg/mL), enrofloxacin (ENRO; 0.12–128 µg/mL), florfenicol (FFN; 0.25–256 µg/mL), oxytetracycline (OXY; 0.5–16 µg/mL), chlortetracycline (CTET; 1–256 µg/mL).

The heat maps in Figure 1 provide an overall impression of how MICs changed by health status and over time. In general, MIC values were lowest for the isolates from healthy cattle and highest for the dead cattle isolates. There was also a trend for MIC values to increase in time. There were also some interesting changes in the MIC values that were derived from the dead cattle isolates for the years 2016–2018. From 2016 onwards, nearly all the isolates were highly resistant to all macrolides. In addition, resistance to ENRO appeared to increase from 2016 to 2018, while the opposite occurred for OXY and CTET.

Table 2 is a crude summary of the MICs from all 211 isolates, ignoring health status, anatomical location, year, and the fact that some animals provided up to three isolates. The macrolide MICs were right censored, with TUL and TYL being both right and left censored. That is, the distributions suggest that the MICs were higher (right censored) or lower (left censored) than the tested range. Due to the right censoring, there was no resolution between the MIC_50_ and MIC_90_ values for the macrolides. The dark vertical line represents the resistance breakpoint for each antimicrobial and where applicableare based on CLSI’s suggested intermediate (I) breakpoints. Most isolates were resistant to all five macrolides.

Statistical analyses were used to compare the MIC distributions of each antimicrobial by health status and production year. Only one isolate (lung) per animal (*n* = 159) was used for these analyses. The lung isolate was chosen because it was the most consistently sampled tissue andbecause of its role in BRD. There were only 19 isolates from diseased cattle, with the mean rank for each antimicrobial generally being between those of the healthy and dead cattle; therefore, this group was omitted for subsequent analyses. In order to assess if the MIC values changed over time, the data displayed in heat maps were analyzed by health status. Theisolates obtained from the nasal cavities of healthy cattle (*n* = 43) were dichotomized into two time-points, “early” (2006–2007) and “late” (2017–2018). A similar manipulation was performed for the MIC data that were obtained from the lung isolates of the dead cattle. In this instance, the “early” isolates were from 2007–2008, while the “late” isolates were derived from cattle that died in the 2017 and 2018 production years. An additional analysis also compared the MIC values from the dead cattle isolates from 2016 versus 2018. These years were chosen because of the changes in the MICs for ENRO, OXY and CTET in these later years.

Table 3 provides the measure of effect (mean ranks) and the level of significance with respect to how the MIC values changed by health status over time. The MIC values that were obtained from the healthy cattle isolates remained relatively constant over a 10 year period, save one exception. The MIC distributions for ENRO changed over time (*p* = 0.004), with the means ranks shifting towards higher MIC values. Analyses of the MICs from the dead cattle isolates, comparing the early and later isolates, found that most of the mean ranks increased over time. The exceptions were FFN, OXY and CTET, whose mean ranks actually dropped; however, only OXY was significantly lower. A similar analysis for the years 2016 versus 2018 found that the MICs for TUL increased over this two-year period, whereas those for FFN, OXY, and CTET decreased.

To summarize, the isolates obtained from the DNP of healthy cattle on arrival to the feedlot did not appreciably change over the 10-year period, with the exception of ENRO. As for the dead cattle isolates, the MICs for the macrolides increased over the 10-year period; the exception was TIL, which had high MICs throughout the 10-year period. Significantly, the mean ranks for FFN, OXY and CTET all decreased in time. This was particularly evident when comparing MICs from the lung isolates of cattle that died in 2016 versus 2018.

Table 4 and Table 5 provide the antimicrobial MIC distributions for the healthy and dead cattle isolates, respectively. A visual inspection found a bimodal distribution of MIC values for TUL, GAM, TYL, and ENRO. The estimated epidemiological cut-off values were TUL 16 µg/mL, GAM 32 µg/mL, TYL 16 µg/mL, and ENRO 0.50 µg/mL.

A Chi-square analysis assessed for differences in the level of resistance (%R) between the healthy and dead cattle isolates. Isolates from the dead cattle were more likely to be resistant to TUL (OR = 11.68; 95% CI 5.03, 27.15; *p* ≤ 0.001), GAM (OR = 5.69; 95% CI 2.32, 13.93; *p* ≤ 0.001), TYL (OR = 3.90; 95% CI 1.37, 11.08; *p* = 0.007), and ENRO (OR = 2.3; 95% CI 1.03, 5.23; *p* = 0.041).

Forty-two DNP isolates from the healthy cattle had corresponding DOF data; one isolate had missing metadata. The data were dichotomized into 0 DOF and ≥14 DOF (range 14–140). The majority (*n* = 25) of the isolates were recovered at 0 DOF, with 17 isolates obtained ≥14 DOF. The mean ranks of the MIC distributions for the isolates from these two populations were similar, with the exception of ENRO (*p* = 0.007, 0 DOF mean rank = 25.27, ≥14 DOF mean rank = 17.00).

The MIC distributions for the macrolides (GAM, TIP, TUL and TYL) were highly correlated, with the exception being TIL (Table 6). The tetracyclines were highly correlated to each other (ρ = 0.895), and moderately correlated to FFN (ρ ≥ 0.653).

As an incidental finding, alamarBlue interfered with OXY at concentrations of ≥32 µg/mL; however, this did not affect the results, since all MICs for OXY were ≤16 µg/mL.

### 3.3. Antimicrobial Susceptibility Testing―Anatomical Region

The MIC distributions for all antimicrobials were compared across 80 paired lung–joint isolates that were derived from 40 animals and 20 isolates from 10 paired lung–DNP isolates. There were no differences in the MIC distributions for any of the antimicrobials between the lung–joint isolates (*p* ≥ 0.124) or the paired lung–DNP isolates (*p* ≥ 0.157).

## 4. Discussion

There are a number of unique features and findings associated with this study. This is the first report of MIC data that were generated from *M. bovis* isolates recovered from western Canadian feedlot cattle over an extended time period. Furthermore, a customized AST panel was developed to test only the antimicrobials that are most commonly used in the metaphylaxis and therapeutic treatment of BRD. This is notable since researchers frequently report using the standard Sensititre Bovine/Porcine MIC Plate (Trek Diagnostics), which tests antimicrobials that are used in both swine and cattle. While the standard plate has utility for conducting AST on multiple different bacterial pathogens, it is not an ideal plate for *M. bovis*. Many of the antimicrobials either have no activity against mycoplasmas (β-lactams and trimethoprim–sulphonamides) or are not routinely used in feedlot cattle (aminoglycosides). Though danofloxacin, a fluoroquinolone, was registered in Canada in 2004 for the treatment of BRD [28], it is used much less than ENRO, so it was not included in the panel. While focusing on fewer antimicrobials allowed for a broader range of serial dilutions, there was still significant right censoring of the MIC values for the macrolides, which is a common finding [19,29,30,31]. This is an issue because the magnitude of resistance and the potential presence of different subpopulations may be missed. Further optimization could be realized by reducing the range of dilutions for ENRO, FFN, OXY and CTET while increasing the upper range of the macrolides.

One of the strengths, but perhaps also a weakness of the study, is the wide-range of isolates recovered from healthy, diseased and dead cattle over multiple years. Sampling in the formative years was designed to answer questions regarding the genetic relatedness of isolates within groups of cattle. Over time, additional samples were obtained to examine how strains varied depending on the anatomical niche they occupied. More recently, increased attention has been given to *M. bovis* and its role in BRD cases that are refractory to standard antimicrobial therapy. Therefore, it is recognized that the purposive and convenience sampling methodologies that were used to acquire the isolates for other research objectives did not constitute a structured random sampling; hence, caution is warranted when interpreting the data. Furthermore, the data were cross-sectional in nature, and, hence, no inference can be made about causality; however, identifying relationships can lead to the generation of hypotheses for future research. That said, important conclusions can be drawn from the data. Specifically, these data highlight the very high levels of antimicrobial resistance to macrolides. This is undoubtedly related to decades of macrolide use in Canadian feedlots. In addition, mycoplasmosis is typically a chronic disease that requires serial courses of therapy, often involving multiple classes of antimicrobials. Thus, significant antimicrobial selection pressure is placed on the strains of *M. bovis* that circulate within a feedlot. While this may explain the very high levels of resistance found in the dead cattle, it is more difficult to reconcile why 100% of the isolates from the 43 healthy cattle were resistant to TIL and TIP.

The finding of high levels of resistance in the healthy cattle was unexpected since most of the cattle would have been sourced from cow–calf operations. This is salient because these animals would have been raised on rangeland during the preceding summer months, where disease and AMU is comparably low in these production settings. However, previous research has shown that *M. bovis* can be recovered from the nasopharyngeal passages and bronchoalveolar lavages of cattle within days of arrival at a feedlot [32]. This may explain the rapid emergence and horizontal transmission of resistant clones within the feedlot, particularly in cattle that were ≥14 DOF. However, this does not account for the level of resistance that was seen in the healthy cattle that were sampled on arrival. Perhaps the clonal spread of resistant bacteria may occur during commingling at auctions and during transport. Consideration must also be given to herd level factors, such as antimicrobial use, which may have selected for resistant strains that circulated within cow–calf operations. Regardless of the reservoir of resistant strains, the MIC data were disconcerting, since, even after controlling for the number of DOF, these healthy cattle arrived at the feedlot harboring *M. bovis* with significant levels of resistance to macrolides. Based on the data, the use of TIP and TIL for treating putative *M. bovis* infections such as CPPS should be avoided. This recommendation is certainly not novel. As early as 2002, Rosenbusch et al. [29] noted that erythromycin and TIL should not be considered for the treatment of *M. bovis* infections. This contention is supported by multiple studies and review articles [33,34,35,36].

While the current study has a number of limitations, the results are quite similar to two previous studies that included AST data from *M. bovis* isolates that were derived from western Canadian feedlot cattle [18,19]. In the first study, Hendrick et al. [18] conducted AST on 51 *M. bovis* isolates that were derived from sick and dead western Canadian feedlot cattle in 2007 and 2008 and reported lower MIC_50_ values than those seen in the current study, which may have been related to the isolates coming from a single feedlot. However, TUL, OXY, CTET and FFN all had MIC_50_ values in the 2–4 µg/mL range, which is comparable to the results reported herein. In both studies, TIL had very high MICs, whereas the isolates from 2007 and 2008 had very low MICs for TUL. These findings can be explained, in part, by AMU. Tilmicosin was registered in Canada in 1990 and, for many years, was the only long-acting macrolide used for BRD, whereas TUL came to the market in the autumn of 2007 [28]. Therefore, very few, if any, of the cattle that were sampled in 2007 and 2008, in either study, would have been treated with TUL. Thus, the heat maps provide a historical perspective on the increasing resistance to TUL over the last decade.

Though there was significant levels of resistance to OXY and CTET, it is important to appreciate that the mean ranks for FFN and the tetracyclines have been decreasing over time. Unlike TUL and TIL, these results cannot be explained by when the products came to market, since FFN was licensed in 1996 and the tetracyclines predate this registration by decades. More likely, the entry of newer macrolides into the marketplace has displaced the use of FFN, OXY and CTET. A study of AMU in western Canadian feedlots found that CTET, which has historically been incorporated into the feed for the control of *Histophilus somni* has been replaced with more efficacious parenterally administered therapies [5]. Further to this reduction, in 2018 the pharmaceutical industry voluntarily removed growth promotion claims from all medically important antimicrobials (MIAs). In addition, Health Canada mandated that, by the end of 2018, all MIAs for veterinary use must be sold by prescription only. These prescribing practices and regulatory changes may account for the lower tetracycline MICs seen in 2018.

A second and more recent western Canadian study reported on the AST results that were generated from 226 *M. bovis* isolates recovered from 211 feedlot cattle that were sampled in the 2014 production year [19]. While many of the data were right censored, the overall results were similar to the current study. Specifically, macrolides had the highest MICs, while ENRO, FFN, OXY and CTET were relatively comparable but with a bias towards slightly higher MICs. These results are relevant because they confirm or validate the results reported herein with respect to the AST findings from the more recently recovered isolates. The slightly higher MICs could be real or may reflect the inherent challenges of comparing AST data across different laboratories. What is clear, however, is that the macrolides had very high levels of resistance, whereas resistance to FFN, ENRO, and the tetracyclines was much lower.

Though the current study was not designed a priori as an AST surveillance study, the results are very consistent with the findings of the two previous studies of western Canadian feedlot cattle [18,19]. In general, all three studies have arrived at the same conclusions. The vast majority of *M. bovis* isolates are resistant to macrolides but susceptible to ENRO, FFN, and OXY. The low level of resistance to ENRO is an important finding since the World Health Organization (WHO) classifies fluoroquinolones as Class I antimicrobials, which are deemed critically important [37]. It is also noteworthy that ENRO AMU has actually been declining in western Canadian feedlots [5], which may explain the low levels of resistance. That said, it was clear from the heat maps that the MICs for ENRO have been increasing in time within both the healthy and dead cattle isolates. Presumably, ENRO is being used when all other treatments have failed to resolve the BRD, resulting in selection pressure on the mycoplasma community. All three studies underscore the need for alternatives to antimicrobials for controlling BRD. Macrolide resistance is very high and is unlikely to change in the near future because most feedlots rely on this class of antimicrobials for the metaphylaxis and treatment of BRD. Potential overuse is compounded by the fact that there is a limited number of therapeutic options, and this may result in increased use of ENRO as a last resort antimicrobial. 

Macrolide resistance has been attributed to single nucleotide polymorphisms (SNPs) or point mutations that are common to all pathogenic mycoplasmas [38,39]. These modifications alter how macrolides interact with the 50S rRNA subunit to inhibit protein synthesis. Specifically, macrolides bind within the 50 s tunnel and interact with the A2058 and A2059 nucleotides [40,41]. It is noteworthy that TIL and TIP are derivatives of TYL, and all three bind at the same site. However, minor changes in how they bind may modify antimicrobial activities [40]. This may explain the lower levels of resistance to TYL versus TIL and TIP in the healthy cattle isolates. Sulyok et al. [35] also reported a cross-resistance between TYL and TIL and speculated, based on similar MIC distributions, that cross-resistance extended to GAM and TUL. Thus, all four macrolides share common point mutations that confer resistance, which explains the highly correlated MICs distributions for GAM, TIP, TUL and TYL. The finding that TIL was not as highly correlated to the other macrolide is a spurious finding. The Spearman correlation compares the distributions of the MICs versus specific values. In the case of TIL, all the isolates essentially had the same MIC (≥256 µg/mL), whereas the other four macrolides had a range of MICs. Thus, the test statistic found that the TIL was not correlated with the others. In this situation, it was best to look at the MIC_50_ and MIC_90_ values, which were identical for all five macrolides for dead cattle isolates. In reality, all the macrolides were indeed highly correlated. This is an important finding because the intense use of macrolides to control and treat BRD [5] invariably leads to an increased AMR [19] in all BRD pathogens. Thus, the results of the current study do not bode well for the continued use of macrolides on a routine basis for the metaphylaxis and therapeutic treatment of mycoplasmosis. There is a compelling argument that macrolides should not be used in animals with chronic BRD, and perhaps even in healthy arrivals to the feedlot, unless the AST data support such therapy.

Macrolide resistance is certainly not unique to this study; rather, this is a growing worldwide phenomenon in human and veterinary medicine. A review article of macrolide resistance of human mycoplasmas noted that *Mycoplasma pneumoniae* has emerged as a major cause of community-acquired pneumonia and, not unlike the current findings, tetracyclines and fluoroquinolones remain effective, whereas macrolide resistance is becoming widespread [38]. As a result, some countries have implemented antimicrobial stewardship programs to curtail macrolide use in humans. Here in Canada, macrolides are classified as Category II drugs; however, the WHO considers them Category I, or highest priority-critically important antimicrobials (HP-CIAs). Furthermore, the WHO states that HP-CIAs should not be used prophylactically or as a first line therapy for animals. Rather, they should only be used if no effective alternative treatment is available. In many instances, macrolides are being administered in feedlots for metaphylaxis and not prophylaxis, with the distinguishing feature being that the cohort of animals being treated is manifesting varying levels of disease. However, data from multiple studies show that OXY or FFN should be considered for first line therapy, ahead of macrolides. This also applies to *Mannheimia haemolytica* and *Pasteurella multocida* [19]. The current state of macrolide resistance in veterinary medicine is a clarion call for developing alternative and sustainable methods for controlling BRD.

Despite the challenges and caveats of extrapolating breakpoints, there is utility in providing veterinary practitioners with a guarded interpretation of the MIC values. A common practice has been to extrapolate the CLSI guidelines from other bacterial members of the BRD complex, specifically *Mannheimia haemolytica*, *Pasteurella multocida*, and *Histophilus somni* [19,28,36,42]. As stated previously, all the CLSI guidelines suggested macrolide breakpoints were adopted with slight modifications. With respect to TIP, the MIC data lacked a bimodal distribution and all MICs were ≥16 µg/mL, suggesting that this population of isolates were non-wild-type. That is, they had acquired some mechanisms of resistance. It also needs to be stressed that the suggested resistant breakpoints encompassed the intermediate breakpoints. The rationale being that isolates in the intermediate interpretative category may require a higher approved dosage of drug [43]. Therefore, from a clinical standpoint, veterinary practitioners want to know which antimicrobial will provide the most successful clinical outcome with a typical dosage regimen.

The same breakpoints were assigned to both OXY and CTET, even though the MIC distributions for CTET were higher than for OXY. This decision was based on a review of MIC data from three other Canadian studies [17,18,19]. In all studies, the MIC values for OXY and CTET were similar, which is understandable since both antimicrobials are impacted by point mutations in the Tet-1 tetracycline binding pocket of the 30S ribosomal unit [35,44]. Until the CLSI establishes interpretative criteria for MIC data for *M. bovis*, researchers and veterinarians will be required to extrapolate from the guidelines for other BRD pathogens. It is worth noting that the CLSI does have guidelines for human mycoplasmas [24], and the suggested resistance breakpoint for tetracycline and *M. hominis* is ≥8 µg/mL; the current study used ≥4 µg/mL, which is a difference of one serial dilution.

Singh et al. [45] reported the first outbreaks of *M. bovis* arthritis in Canadian calves in 1971; however, it was Haines et al. in 2001 who found *M. bovis* in 71% of lungs and 45% of joints of western Canadian feedlot cattle that failed to respond to antimicrobial therapy [46]. Despite a growing body of evidence pointing to the role of *M. bovis* in chronic BRD cases in Canadian feedlot cattle [7,10], AST is not typically performed. Rather, feedlot veterinarians have historically been more concerned with *M. haemolytica*, *Pastuerella multicoda*, and *Histophilus somni*, which together account for the majority of early arrival peracute to acute cases of BRD. Thus, the choice of antimicrobials is determined by algorithms that take the level of risk assigned to a cohort of animals arriving at the feedlot into account. Others, however, have posited that AST should be performed when animals fail to respond to multiple classes of antimicrobials [34,47]. The question then becomes how often and how many isolates are needed, as well as from what animals (healthy, sick or dead). Previous research has shown that during BRD outbreaks, a single dominant clone of *M. bovis* will emerge and spread within a pen [48]. However, in large scale studies of *M. pneumoniae* associated with endemics and epidemics of human mycoplasmosis, the infections were polyclonal [49]. Therefore, in large feedlots, multiple clones, rather than a single clone, may be spreading and infecting cattle. This has implications in respect to AST, AMR, AMU, and vaccine development.

Another interesting feature of this study was the analyses of the paired lung–joint and lung–DNP isolates, which found no differences in the MIC distributions for any antimicrobial. Previously, researchers noted that respiratory tract isolates had a higher MIC_50_ for TUL compared to isolates from the lung, milk, and synovial fluids [50]. In another study, *M. bovis* isolates from milk samples had higher MICs than did those from the lungs for spectinomycin, though not for other antimicrobials [51]. However, in both studies, the samples were not paired, but isolates came from samples submitted to diagnostic laboratories. The current study examined the AST profiles of isolates that were obtained from the same animals. Whether these isolates represent a single clone that colonizes the DNP and then gains entry to the lung and spreads hematogenously to the joints and other tissues is unknown. Molecular techniques should be able to answer whether the isolates in the DNP, lungs and joints are mono or polyclonal.

## 5. Conclusions

*Mycoplasma bovis* is an important pathogen of the bovine respiratory disease complex. While the colonization of the respiratory tract occurs within days, cattle with mycoplasmosis often linger in the feedlot for many weeks, during which time they receive multiple courses of antimicrobial therapy. Additionally, high-risk cattle are routinely given macrolides for metaphylaxis. As a result, there is widespread antimicrobial resistance to the five most commonly used macrolides. The challenges facing the feedlot sector is that this class of antimicrobials is extensively used for the prevention and treatment of all causes of BRD. As a result, the resistance issue is not unique to mycoplasmas, but it is a cause for concern in the management of BRD in general. One potential solution would be to rotate macrolides as first-choice treatment to second or third choice, elevating the use of OXY and FFN. However, this is not a long-term solution; rather, a paradigm shift is needed if the industry is to remain sustainable. It also needs to be stated that AMR is not just an economic issue. Chronic mycoplasmosis leads to pain and suffering, especially if the cattle have CPPS. Therefore, AMR is very much an animal welfare issue.

## Figures and Tables

**Table 1:** Cross tabulation of 211 *Mycoplasma bovis* isolates by sample (anatomical region or health status) and production year.

Sample	Feedlot Production Year								
	2006	2007	2008	2014	2015	2016	2017	2018	Total
Anatomical Region									
DNP	12	27	1	0	0	0	14	24	78
Lung	0	2	6	7	11	19	20	19	84
Joint	0	1	1	2	2	16	21	6	49
Total	12	30	8	9	13	35	55	49	211
Health Status									
Healthy	4	14	0	0	0	0	14	11	43
Diseased	8	11	0	0	0	0	0	0	19
Dead	0	5	8	9	13	35	41	38	149
Total	12	30	8	9	13	35	55	49	211

**Table 2:** Antibiotic susceptibility test results of the 211 *Mycoplasma bovis* isolates that were recovered from 159 healthy, diseased, and dead cattle between 2006 and 2018.

Antibiotic	Class	≤0.12	0.25	0.50	1	2	4	8	16	32	64	128	≥256	%R	MIC Range (µg/mL)	MIC_50_ (µg/mL)	MIC_90_ (µg/mL)
Tulathromycin	Macrolide		52	2	2	1	2	2		3	4	25	118	71.1	≤0.25–≥256	≥256	≥256
Gamithromycin	Macrolide				5	17	17	9	3	1	6	3	150	81.5	1–≥256	≥256	≥256
Tilmicosin	Macrolide					1					2	6	202	99.5	2–≥256	≥256	≥256
Tildipirosin	Macrolide							1	8	13	20	169		100	8–≥128	≥128	≥128
Tylosin	Macrolide				3	10	13	22	6	12	11	134		87.7	1–≥128	≥128	≥128
Enrofloxacin	Fluoroquinolone	120	15			20	30	19	7					36.0	≤0.12–16	0.25	4
Florfenicol	Phenicol		2	18	72	100	18	1						9.0	≤0.25–8	2	2
Oxytetracycline	Tetracycline			6	30	75	85	14	1					47.4	0.50–16	2	4
Chlortetracycline	Tetracycline				9	31	62	74	32	3				81.0	≤1–32	8	16

The dark vertical lines denote the resistance breakpoint, whereas the shaded cells are antimicrobial concentrations that were not tested.

**Table 3:** Summary of the Mann–Whitney U-test showing the change in mean ranks for each antimicrobial when analyzed by health status (healthy or dead) and over time.

Sample	TUL	GAM	TIL	TIP	TYL	ENRO	FFN	OXY	CTET
Healthy (N = 43)									
2006–2007 (*n* = 18)	24.39	21.31	21.17	18.61	21.83	16.92	19.64	22.67	22.86
2017–2018 (*n* = 25)	20.28	22.50	22.60	24.44	22.12	25.66	23.70	21.52	21.38
	*p* = 0.225	*p* = 0.753	*p* = 0.403	*p* = 0.091	*p* = 0.939	*p* = 0.004	*p* = 0.206	*p* = 0.745	*p* = 0.676
Dead (N = 47)									
2007–2008 (*n* = 8)	7.31	6.94	24.00	9.38	4.56	10.50	31.88	32.50	29.13
2017–2018 (*n* = 39)	27.42	27.50	24.00	27.00	27.99	26.77	22.38	22.26	22.95
	*p* < 0.001	*p* < 0.001	*p* = 1.000	*p* < 0.001	*p* < 0.001	*p* = 0.001	*p* = 0.060	*p* = 0.044	*p* = 0.231
Dead (N = 38)									
2016 (*n* = 19)	14.84	18.00	19.50	19.00	17.61	18.08	25.61	25.16	25.97
2018 (*n* = 19)	24.16	21.00	19.50	20.00	21.39	20.92	13.39	13.84	13.03
	*p* = 0.002	*p* = 0.075	*p* = 1.000	*p* = 0.317	*p* = 0.097	*p* = 0.413	*p* < 0.001	*p* = 0.001	*p* < 0.001

**Table 4:** Antibiotic susceptibility test results of 43 *Mycoplasma bovis* isolates that were recovered from the nasopharyngeal passages of healthy cattle between 2006 and 2018.

Antibiotic	Class	≤0.12	0.25	0.50	1	2	4	8	16	32	64	128	≥256	%R	MIC Range (µg/mL)	MIC_50_ (µg/mL)	MIC_90_ (µg/mL)
Tulathromycin	Macrolide		26	1	1		2			1	1		11	30.2	≤0.25–≥256	≤0.25	≥256
Gamithromycin	Macrolide					8	9	7	2	1	3		13	60.5	2–≥256	8	≥256
Tilmicosin	Macrolide										1	2	40	100	64–≥256	≥256	≥256
Tildipirosin	Macrolide								2	8	8	25		100	16–≥128	≥128	≥128
Tylosin	Macrolide					3	7	15	4	4		10		76.7	2–≥128	8	≥128
Enrofloxacin	Fluoroquinolone	31	2				1	5	4					23.3	≤0.12–16	≤0.12	8
Florfenicol	Phenicol			1	8	29	5							11.6	0.5–4	2	4
Oxytetracycline	Tetracycline			3	1	12	23	4						62.8	≤0.5–8	4	4
Chlortetracycline	Tetracycline				3	2	10	23	5					88.4	≤1–16	8	16

The dark vertical lines denote the resistance breakpoint, whereas the shaded cells are antimicrobial concentrations that were not tested.

**Table 5:** Antibiotic susceptibility test results for 97 *Mycoplasma bovis* isolates that were recovered from the lungs of dead cattle between 2007 and 2018 via the broth microdilution method.

Antibiotic	Class	≤0.12	0.25	0.50	1	2	4	8	16	32	64	128	≥256	%R	MIC Range (µg/mL)	MIC_50_ (µg/mL)	MIC_90_ (µg/mL)
Tulathromycin	Macrolide		13	1	1			1			1	20	60	83.5	≤0.25–≥256	≥256	≥256
Gamithromycin	Macrolide				1	4	5	1	1		2	1	82	89.7	1–≥256	≥256	≥256
Tilmicosin	Macrolide					1						1	95	99.0	2–≥256	≥256	≥256
Tildipirosin	Macrolide								2	2	7	86		100	16–≥128	≥128	≥128
Tylosin	Macrolide				1	4	2	4	2	7	6	71		92.8	≤1–≥128	≥128	≥128
Enrofloxacin	Fluoroquinolone	47	10			13	18	6	3					41.2	≤0.12–16	0.25	4
Florfenicol	Phenicol		2	10	33	44	7	1						8.2	≤0.25–8	2	2
Oxytetracycline	Tetracycline			3	13	37	36	7	1					45.4	≤0.5–≥16	2	4
Chlortetracycline	Tetracycline				4	15	27	34	15	2				80.4	≤1–32	8	16

The dark vertical lines denote the resistance breakpoint, whereas the shaded cells are antimicrobial concentrations that were not tested.

**Table 6:** Correlation of the minimum inhibitory concentrations (MICs) from 140 *M. bovis* isolates for nine different antimicrobials.

	ENRO	TIP	GAM	TUL	FFN	OXY	CTET	TYL	TIL
ENRO		0.166 *	0.262 **	0.295 **	−0.357 **	−0.170 *	−0.112	0.389 **	0.163
TIP	0.166 *		0.846 **	0.690 **	−0.027	−0.091	−0.047	0.720 **	0.336 **
GAM	0.262 **	0.846 **		0.874 **	−0.183 *	−0.139	−0.097	0.876 **	0.333 **
TUL	0.295 **	0.690 **	0.874 **		−0.255 **	−0.187 *	−0.145	0.838 **	0.238 **
FFN	−0.357 **	−0.027	−0.183 *	−0.255 **		0.694 **	0.653 **	−0.225 **	0.085
OXY	−0.170 *	−0.091	−0.139	−0.187 *	0.694 **		0.895 **	−0.102	0.215 *
CTET	−0.112	−0.047	−0.097	‒0.145	0.653 **	0.895 **		−0.018	0.171 *
TYL	0.389 **	0.720 **	0.876 **	0.838 **	−0.225 **	−0.102	−0.018		0.335 **
TIL	0.163	0.336 **	0.333 **	0.238 **	0.085	0.215 *	0.171 *	0.335 **	

** Correlation is significant at the 0.01 level (2-tailed). * Correlation is significant at the 0.05 level (2-tailed).

## Appendix A

**Table:**

Year	Feedlot	Cohort	Isolate ID	Animal #	Anatomical Region	Health Status	DOF
2006	F6	C13	MPLM0648	1	DNP	Diseased	90
			MPLM0654	2	DNP	Diseased	90
	F14	C21	MPLM0649	3	DNP	Diseased	90
			MPLM0786	4	DNP	Diseased	90
	F16	C23	MPLM0845	5	DNP	Healthy	
	F20	C29	MPLM0810	6	DNP	Diseased	150
			MPLM0642	7	DNP	Diseased	150
			MPLM0651	8	DNP	Diseased	150
	F30	C40	MPLM0643	9	DNP	Healthy	140
			MPLM0811	10	DNP	Healthy	14
			MPLM0813	11	DNP	Diseased	
			MPLM0640	12	DNP	Healthy	14
	**N = 5**	**N = 5**	**N = 12**	**N = 12**			
2007	F1	C1	MPLM0788	13	Lung	Dead	47
	F8	C15	MPLM0632	14	DNP	Dead	
	F9	C16	MPLM0795	15	Lung	Dead	
			MPLM0631		Joint	Dead	60
	F15	C22	MPLM0664	16	DNP	Diseased	15
			MPLM0660	17	DNP	Diseased	0
			MPLM0665	18	DNP	Diseased	21
			MPLM0644	19	DNP	Diseased	21
			MPLM0669	20	DNP	Dead	41
			MPLM0661	21	DNP	Diseased	12
			MPLM0647	22	DNP	Diseased	16
			MPLM0657	23	DNP	Diseased	45
			MPLM0713	24	DNP	Healthy	15
			MPLM0714	25	DNP	Healthy	15
			MPLM0684	26	DNP	Healthy	15
			MPLM0652	27	DNP	Diseased	10
			MPLM0662	28	DNP	Diseased	21
			MPLM0700	29	DNP	Healthy	15
			MPLM0703	30	DNP	Healthy	15
			MPLM0715	31	DNP	Healthy	15
			MPLM0706	32	DNP	Healthy	15
			MPLM0645	33	DNP	Diseased	10
			MPLM0666	34	DNP	Diseased	10
			MPLM0692	35	DNP	Healthy	15
			MPLM0698	36	DNP	Healthy	15
	F16	C24	MPLM0670	37	DNP	Healthy	14
			MPLM0671	38	DNP	Healthy	14
		C25	MPLM0667	39	DNP	Healthy	90
			MPLM0668	40	DNP	Healthy	90
			MPLM0646	41	DNP	Healthy	90
	**N = 5**	**N = 6**	**N = 30**	**N = 29**			
2008	F7	C14	MPLM0781	42	Lung	Dead	
			MPLM0602		DNP	Dead	60
			MPLM0784	43	Lung	Dead	
	F8	C15	MPLM0630	44	Joint	Dead	30
	F22	C31	MPLM0789	45	Lung	Dead	
			MPLM0792	46	Lung	Dead	
	F23	C32	MPLM0783	47	Lung	Dead	
			MPLM0790	48	Lung	Dead	
	**N = 4**	**N = 4**	**N = 8**	**N = 7**			
2014	F2	C5	MYCO066	49	Lung	Dead	35
			MYCO096	50	Lung	Dead	96
	F3	C6	MP0219	51	Lung	Dead	39
			MP0209		Joint	Dead	39
	F13	C20	MYCO081	52	Joint	Dead	31
	F17	C26	MP0064	53	Lung	Dead	193
	F19	C28	MPLM0636	54	Lung	Dead	93
	F21	C30	MYCO062	55	Lung	Dead	76
			MYCO076	56	Lung	Dead	24
	**N = 6**	**N = 6**	**N = 9**	**N = 8**			
2015	F3	C7	MP0071	57	Lung	Dead	42
			MP0063	58	Lung	Dead	42
			MPLM0637	59	Lung	Dead	31
			MP0058	60	Lung	Dead	31
			MP0073	61	Lung	Dead	67
			MP0077	62	Lung	Dead	61
			MP0079	63	Lung	Dead	67
			MPLM0638		Joint	Dead	67
			MP0183	64	Joint	Dead	217
	F11	C18	MPLM0634	65	Lung	Dead	
	F13	C20	MP0057	66	Lung	Dead	80
	F21	C30	MP0070	67	Lung	Dead	20
	F27	C37	MP0075	68	Lung	Dead	46
	**N = 5**	**N = 5**	**N = 13**	**N = 12**			
2016	F1	C2	MPLM0041	69	Lung	Dead	46
			MPLM0042		Joint	Dead	46
			MPLM0033	70	Lung	Dead	48
			MPLM0034		Joint	Dead	48
			MPLM0029	71	Lung	Dead	
			MPLM0030		Joint	Dead	
	F18	C27	MPLM0021	72	Lung	Dead	55
			MPLM0022		Joint	Dead	55
			MPLM0009	73	Lung	Dead	22
			MPLM0010		Joint	Dead	22
			MPLM0013	74	Lung	Dead	26
			MPLM0014		Joint	Dead	26
			MPLM0025	75	Lung	Dead	15
			MPLM0026		Joint	Dead	15
			MPLM0037	76	Lung	Dead	81
			MPLM0017	77	Lung	Dead	43
			MPLM0018		Joint	Dead	43
			MPLM0015	78	Lung	Dead	31
			MPLM0016		Joint	Dead	31
			MPLM0007	79	Lung	Dead	9
			MPLM0008		Joint	Dead	9
			MPLM0035	80	Lung	Dead	38
			MPLM0036		Joint	Dead	38
			MPLM0031	81	Lung	Dead	34
			MPLM0019	82	Lung	Dead	55
			MPLM0020		Joint	Dead	55
			MPLM0011	83	Lung	Dead	22
			MPLM0012		Joint	Dead	22
			MPLM0039	84	Lung	Dead	50
			MPLM0040		Joint	Dead	50
	F19	C28	MPLM0134	85	Lung	Dead	60
			MPLM0135		Joint	Dead	60
			MPLM0132	86	Lung	Dead	30
	F28	C38	MPLM0003	87	Lung	Dead	28
			MPLM0004		Joint	Dead	28
	**N = 4**	**N = 4**	**N = 35**	**N = 19**			
2017	F1	C3	MPLM0114	88	Lung	Dead	33
			MPLM0102	89	Lung	Dead	40
			MPLM0103		Joint	Dead	40
			MPLM0160	90	Lung	Dead	52
			MPLM0093	91	Lung	Dead	37
			MPLM0094		Joint	Dead	37
			MPLM0090	92	Lung	Dead	45
			MPLM0091		Joint	Dead	45
			MPLM0111	93	Lung	Dead	17
			MPLM0112		Joint	Dead	17
			MPLM0087	94	Lung	Dead	60
			MPLM0088		Joint	Dead	60
			MPLM0105	95	Lung	Dead	54
			MPLM0106		Joint	Dead	54
			MPLM0064	96	Joint	Dead	
			MPLM0164	97	Joint	Dead	30
			MPLM0143	98	Joint	Dead	24
			MPLM0066	99	Lung	Dead	
			MPLM0067		Joint	Dead	
			MPLM0108	100	Lung	Dead	44
			MPLM0145	101	Lung	Dead	42
			MPLM0146		Joint	Dead	42
			MPLM0154	102	Lung	Dead	40
			MPLM0155		Joint	Dead	40
			MPLM0073	103	Joint	Dead	
			MPLM0157	104	Lung	Dead	46
			MPLM0158		Joint	Dead	46
			MPLM0084	105	Lung	Dead	33
			MPLM0085		Joint	Dead	33
			MPLM0148	106	Lung	Dead	48
			MPLM0149		Joint	Dead	48
			MPLM0069	107	Lung	Dead	
			MPLM0070		Joint	Dead	
	F4	C8	MPLM0831	108	DNP	Healthy	0
	F5	C10	MPLM0833	109	DNP	Healthy	0
			MPLM0834	110	DNP	Healthy	0
			MPLM0837	111	DNP	Healthy	0
			MPLM0838	112	DNP	Healthy	0
		C11	MPLM0832	113	DNP	Healthy	0
			MPLM0835	114	DNP	Healthy	0
		C12	MPLM0839	115	DNP	Healthy	0
	F10	C17	MPLM0081	116	Lung	Dead	180
	F12	C19	MPLM0167	117	Joint	Dead	
			MPLM0057	118	Lung	Dead	
			MPLM0058		Joint	Dead	
			MPLM0060	119	Lung	Dead	
			MPLM0061		Joint	Dead	
			MPLM0054	120	Lung	Dead	
			MPLM0076	121	Joint	Dead	
	F24	C33	MPLM0815	122	DNP	Healthy	0
			MPLM0819	123	DNP	Healthy	0
			MPLM0820	124	DNP	Healthy	0
	F26	C36	MPLM0821	125	DNP	Healthy	0
			MPLM0822	126	DNP	Healthy	0
	F29	C39	MPLM0826	127	DNP	Healthy	0
	**N = 8**	**N = 10**	**N = 55**	**N = 40**			
2018	F1	C4	MPLM0563	128	Lung	Dead	
			MPLM0533	129	Lung	Dead	65
			MPLM0534		Joint	Dead	65
			MPLM0535		DNP	Dead	65
			MPLM0569	130	Lung	Dead	27
			MPLM0552	131	Lung	Dead	36
			MPLM0553		DNP	Dead	36
			MPLM0611	132	Lung	Dead	15
			MPLM0622	133	DNP	Dead	33
			MPLM0566	134	Lung	Dead	30
			MPLM0567		DNP	Dead	30
			MPLM0537	135	Lung	Dead	27
			MPLM0538		Joint	Dead	27
			MPLM0539		DNP	Dead	27
			MPLM0593	136	Lung	Dead	44
			MPLM0555	137	Lung	Dead	60
			MPLM0556		Joint	Dead	60
			MPLM0549	138	Lung	Dead	34
			MPLM0587	139	Lung	Dead	34
			MPLM0588		DNP	Dead	34
			MPLM0584	140	Lung	Dead	44
			MPLM0624	141	Lung	Dead	33
			MPLM0625		DNP	Dead	33
			MPLM0541	142	Lung	Dead	71
			MPLM0542		Joint	Dead	71
			MPLM0543		DNP	Dead	71
			MPLM0545	143	Lung	Dead	56
			MPLM0546		Joint	Dead	56
			MPLM0547		DNP	Dead	56
			MPLM0608	144	Lung	Dead	61
			MPLM0609		DNP	Dead	61
			MPLM0582	145	DNP	Dead	61
			MPLM0559	146	Lung	Dead	33
			MPLM0560		Joint	Dead	33
			MPLM0578	147	Lung	Dead	15
			MPLM0579		DNP	Dead	15
	F4	C9	MPLM0827	148	DNP	Healthy	0
			MPLM0828	149	DNP	Healthy	0
			MPLM0829	150	DNP	Healthy	0
			MPLM0830	151	DNP	Healthy	0
	F24	C34	MPLM0816	152	DNP	Healthy	0
			MPLM0817	153	DNP	Healthy	0
			MPLM0818	154	DNP	Healthy	0
	F25	C35	MPLM0627	155	Lung	Dead	
			MPLM0628		DNP	Dead	
	F29	C40	MPLM0823	156	DNP	Healthy	0
			MPLM0824	157	DNP	Healthy	0
			MPLM0825	158	DNP	Healthy	0
	F31	C12	MPLM0836	159	DNP	Healthy	0
	**N = 6**	**N = 6**	**N = 49**	**N = 32**			
